# The herbicide triflusulfuron-meth­yl

**DOI:** 10.1107/S1600536811023166

**Published:** 2011-06-22

**Authors:** Kurt Mereiter

**Affiliations:** a Institute of Chemical Technologies and Analytics, Vienna University of Technology, Getreidemarkt 9/164SC, A-1060 Vienna, Austria

## Abstract

The mol­ecule of the title compound [systematic name: methyl 2-({[4-dimethyl­amino-6-(2,2,2-trifluoro­eth­oxy)-1,3,5-triazin-2-yl]carbamo­yl}sulfamo­yl)-3-methyl­benzoate], C_17_H_19_F_3_N_6_O_6_S, features a nearly planar (r.m.s. deviation = 0.098 Å) dimethyl­amino­triazinyl-urea group with a short intra­molecular N—H⋯N hydrogen bond to a triazine N atom. An intra­molecular dipole–dipole inter­action between the sulfamide and carboxyl­ate groups, with O_s_⋯C_c_ = 2.800 (1) Å and N_s_⋯O_c_ = 2.835 (1) Å, controls the orientation of the methyl­benzoate group and the shape of the mol­ecule. The crystal structure is stabilized by inter­molecular N—H⋯N hydrogen bonding, C—H⋯*X* (*X* = N,O) inter­actions and arene π–π stacking.

## Related literature

For general information on the synthesis and herbicidal properties of the title compound, see: EFSA (2008 ▶); Moon (1989 ▶); Peeples *et al.* (1991 ▶); Wittenbach *et al.* (1994 ▶). For the inhibition mechanism of sulfonyl­urea herbicides on acetohy­droxy acid syntheases, see: Duggleby *et al.* (2008 ▶); McCourt *et al.* (2005 ▶). For the crystal structures of related sulfonyl­urea compounds, see: Ma, Wang *et al.* (2003 ▶); Ma, Li *et al.* (2003 ▶); Wang *et al.* (2004 ▶); Sorokin *et al.* (1993 ▶); Liu *et al.* (2008 ▶). For a description of the Cambridge Structural Database, see: Allen (2002 ▶). For bond-length data, see: Allen *et al.* (1987 ▶).
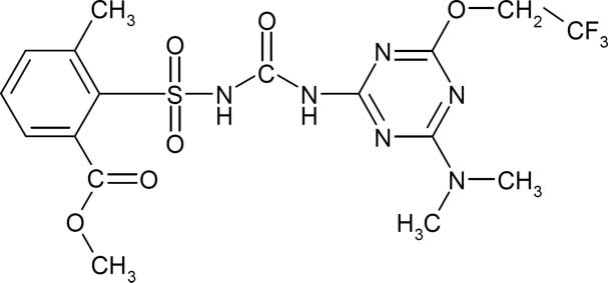


## Experimental

### 

#### Crystal data

C_17_H_19_F_3_N_6_O_6_S
*M* *_r_* = 492.44
Monoclinic, 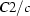

*a* = 16.7107 (11) Å
*b* = 15.6406 (11) Å
*c* = 17.1875 (12) Å
β = 107.035 (1)°
*V* = 4295.1 (5) Å^3^
*Z* = 8
Mo *K*α radiation
μ = 0.23 mm^−1^
*T* = 100 K
0.55 × 0.35 × 0.30 mm

#### Data collection

Bruker Kappa APEXII CCD diffractometer
Absorption correction: multi-scan (*SADABS*; Bruker, 2008 ▶) *T*_min_ = 0.84, *T*_max_ = 0.94
31010 measured reflections
6217 independent reflections
5708 reflections with *I* > 2σ(*I*)
*R*_int_ = 0.024

#### Refinement

*R*[*F* ^2^ > 2σ(*F* ^2^)] = 0.035
*wR*(*F* ^2^) = 0.102
*S* = 1.07
6217 reflections
302 parameters
H-atom parameters constrained
Δρ_max_ = 0.50 e Å^−3^
Δρ_min_ = −0.49 e Å^−3^

### 

Data collection: *APEX2* (Bruker, 2008 ▶); cell refinement: *SAINT* (Bruker, 2008 ▶); data reduction: *SAINT*, *SADABS* and *XPREP* (Bruker, 2008 ▶); program(s) used to solve structure: *SHELXS97* (Sheldrick, 2008 ▶); program(s) used to refine structure: *SHELXL97* (Sheldrick, 2008 ▶); molecular graphics: *Mercury* (Macrae *et al.*, 2006 ▶); software used to prepare material for publication: *PLATON* (Spek, 2009 ▶) and *publCIF* (Westrip, 2010 ▶).

## Supplementary Material

Crystal structure: contains datablock(s) I, global. DOI: 10.1107/S1600536811023166/gk2384sup1.cif

Structure factors: contains datablock(s) I. DOI: 10.1107/S1600536811023166/gk2384Isup2.hkl

Supplementary material file. DOI: 10.1107/S1600536811023166/gk2384Isup3.cml

Additional supplementary materials:  crystallographic information; 3D view; checkCIF report

## Figures and Tables

**Table 1:** Hydrogen-bond geometry (Å, °)

*D*—H⋯*A*	*D*—H	H⋯*A*	*D*⋯*A*	*D*—H⋯*A*
N1—H1*N*⋯N5	0.88	1.95	2.6410 (13)	135
N2—H2*N*⋯N3^i^	0.88	2.03	2.8998 (12)	172
C9—H9*A*⋯O5	0.98	2.42	3.2253 (16)	139
C14—H14*B*⋯O3	0.98	2.59	3.4363 (16)	145
C14—H14*C*⋯O5^ii^	0.98	2.42	3.2637 (14)	143
C16—H16*A*⋯O2^iii^	0.99	2.55	3.4209 (14)	147
C16—H16*B*⋯O1^iv^	0.99	2.32	3.2325 (14)	153

Symmetry codes: (i) 
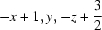
; (ii) 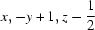; (iii) 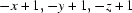; (iv) 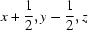.

**Fig. 1.**
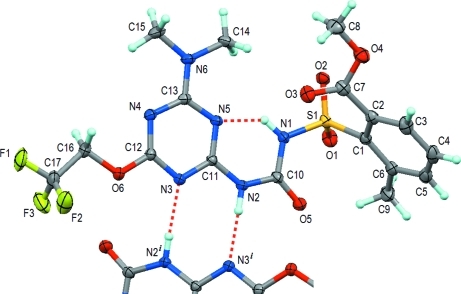
The molecular structure of (I) with displacement ellipsoids for the non-hydrogen atoms drawn at the 50% probability level. Hydrogen bonds are drawn as dashed red lines. Symmetry code i: -x + 1, y, -z + 3/2. Intramolecular interactions are N1···N5 = 2.641 (1) Å, O2···C7 = 2.800 (1) Å, and O3···N1 = 2.835 (1) Å.

**Fig. 2.**
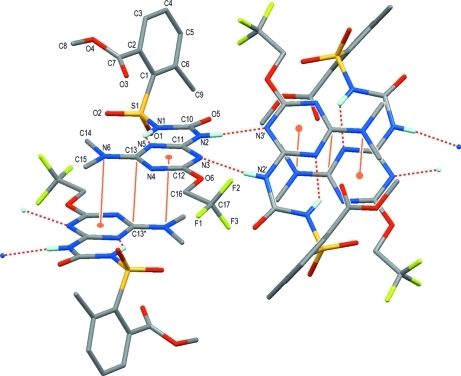
View of the crystal structure of (I) showing N—H···N hydrogen bonds as dashed red lines and π-π stacking interactions between dimethylamino-triazine groups as orange lines. C-bonded H-atoms omitted for clarity.

**Fig. 3.**
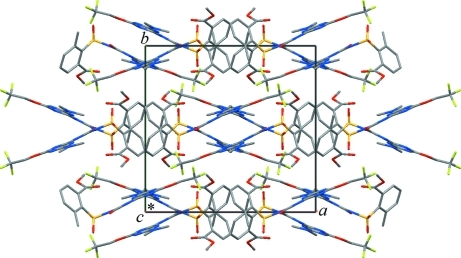
Packing diagram of (I) in a view down the c-axis, i.e. approximately along the intermolecular hydrogen bonds N2—H2n···N3. H-atoms omitted for clarity.

## supplementary crystallographic information

### Comment

Triflusulfuron-methyl, methyl 2-[[4-(dimethylamino)-6-(2,2,2-trifluoroethoxy)-1,3,5-triazin-2-yl]carbamoylsulfamoyl]-3-methylbenzoate, (I), is the methyl ester of Triflusulfuron. Both substances are triazinylsulfonylurea herbicides, a subclass of sulfonylurea herbicides. These substances are efficient inhibitors of acetohydroxyacid synthase (AHAS), which impairs the synthesis of the branched-chain amino acids valine, leucine and isoleucine in plants leading to the cessation of cell division and subsequent growth processes (EFSA, 2008). Triflusulfuron-methyl is in use as a post-emergence herbicide in crop protection of sugar and fodder beet in order to control various annual grasses and broad–leaved weeds like fools parsley, mayweeds, brassica species, or small nettle (Moon, 1989; Peeples *et al.*, 1991; Wittenbach *et al.*, 1994). Trade names of the originator DuPont^TM^ (Moon, 1989) for formulations of (I) are UpBeet in the USA and Canada, and Debut or Safari in Europe. For mammalians, the toxicity of Triflusulfuron-methyl is comparatively low whereas it is high for aquatic organisms (EFSA, 2008). In view of the actual importance of this substance a crystal structure determination was carried out and is presented here.

A view of the asymmetric unit of the structure is presented in Fig. 1. Bond lengths and angles in the molecule exhibit normal values (Allen *et al.*, 1987) and are in good agreement with the few related crystal structures, which have been determined so far with comparable accuracy [refcodes RACCOO (Ma, Wang *et al.*, 2003), XUYTUG (Ma, Li *et al.*, 2003), NAFWAT (Wang *et al.*, 2004) and YUZHUW (Sorokin *et al.*, 1993); Cambridge Structural Database, Version 5.31, with Aug. 2010 updates; Allen, 2002]. The molecule of the title compound can be divided into three approximately planar parts: (i) the triazinylsulfonylurea group including the triazine substituents N(CH_3_)_2_ and OCH_2_CF_3_ comprising the 18 atoms S1, O2, O5, O6, N1 to N6, and C10 to C17 with a r.m.s. deviation from planarity of 0.098 Å; (ii) the methylphenyl group with a r.m.s. deviation from planarity of 0.011 Å; and (iii) the methylcarboxylate group with a r.m.s. deviation from planarity of 0.002 Å. The interplanar angle is 75.26 (2)° between (i) and (ii), and 48.07 (6)° between (ii) and (iii). The mutual orienations of these three parts and the shape of the entire molecule are controlled by two remarkable intramolecular interactions. Firstly, by a short and strong internal hydrogen bond N1—H1n···N5 with N1···N5 = 2.641 (1) Å, where H1n is the most acidic hydrogen atom of the compound and N5 is a triazine nitrogen as the acceptor (Fig. 1). Secondly, by an intramolecular dipole-dipole interaction between the sulfamoyl and the carboxylate group with the very short contact distances O2···C7 = 2.800 (1) Å and O3···N1 = 2.835 (1) Å (Fig. 1). These interactions are characteristic also for other triazinyl- and pyrimidinyl-sulfonylurea compounds, *e.g.* for RACCOO (Ma, Wang *et al.*, 2003) where the corresponding distances are N(H)···N = 2.690 Å, O···C = 2.823 Å, and O···N = 3.198 Å, respectively. As the result the stereochemistry and shape of the molecule cores of all these compounds is in crystalline state remarkably uniform. Interestingly this stereochemistry changes drastically when the molecules enter the herbicide binding site of acetohydroxyacid synthases (AHAS): There molecules like (I) convert by ~180°-rotations about the bonds C11—N2 and N2—C10, which opens the intramolecular hydrogen bond N1—H1n···N5, makes both NH groups to be approximately parallel and *exo*-oriented toward hydrogen bond acceptors like H_2_O, and brings O5, N3, and O6 closely together for hydrogen bond interactions with the guanidine terminus of an arginine in the herbicide pocket of AHAS enzymes (McCourt *et al.*, 2005; Duggleby *et al.*, 2008). Such conversion also takes place under concomitant deprotonation of N1 when a triazinyl- or pyrimidinyl-sulfonylurea compound chelates a metal atom with the urea oxygen (O becomes formally anionic by NH-deprotonation) and a triazine/pyrimidine nitrogen, *e.g.* in the Cu complex MOGTIM (Liu *et al.*, 2008).

Further hydrogen bond-like interactions in (I) are listed in Table 1. The most important of them, apart from N1—H1n···N5, is the intermolecular bond N2—H2n···N3^i^, two of which link a pair of molecules related by a twofold symmetry axis (Fig. 1). In the crystal lattice these hydrogen bonded pairs interact then by π-π-stacking between pairs of inversion related and mutually slipped dimethylamino-triazine groups with a C13—C13(1 - *x*,1 - *y*,1 - *z*) distance of 3.460 (2) Å (ring-ring perpendicular distance 3.420 Å, ring-ring slippage 2.743 Å) and by weaker π-π-stacking between inversion related pairs of benzene rings (perpendicular distance 3.587 Å, ring-ring slippage 1.002 Å). Fig. 2 exemplifies a part of the crystal lattice with N—H···N hydrogen bonds and triazine π-π- interactions. A packing diagram of the structure is depicted in Fig. 3.

### Experimental

A sample of (I) was obtained from Sigma-Aldrich. It was recrystallized from methanol by room temperature evaporation to furnish colourless prisms suitable for X-ray diffraction.

### Refinement

H atoms were located in a difference Fourier map, placed in calculated positions and thereafter treated as riding. A torsional parameter was refined for each methyl group. *U*_iso_(H) = 1.2*U*_eq_(C,N) for CH, CH_2_ and NH groups; *U*_iso_(H) = 1.5*U*_eq_(C) for CH_3_ groups.

### Crystal data

**Table:**

C_17_H_19_F_3_N_6_O_6_S	*F*(000) = 2032
*M**_r_* = 492.44	*D*_x_ = 1.523 Mg m^−^^3^
Monoclinic, *C*2/*c*	Mo *K*α radiation, λ = 0.71073 Å
*a* = 16.7107 (11) Å	Cell parameters from 9962 reflections
*b* = 15.6406 (11) Å	θ = 2.6–30.5°
*c* = 17.1875 (12) Å	µ = 0.23 mm^−^^1^
β = 107.035 (1)°	*T* = 100 K
*V* = 4295.1 (5) Å^3^	Block, colourless
*Z* = 8	0.55 × 0.35 × 0.30 mm

### Data collection

**Table:**

Bruker Kappa APEXII CCD diffractometer	6217 independent reflections
Radiation source: fine-focus sealed tube	5708 reflections with *I* > 2σ(*I*)
graphite	*R*_int_ = 0.024
φ and ω scans	θ_max_ = 30.0°, θ_min_ = 2.9°
Absorption correction: multi-scan (*SADABS*; Bruker, 2008)	*h* = −23→23
*T*_min_ = 0.84, *T*_max_ = 0.94	*k* = −22→22
31010 measured reflections	*l* = −23→23

### Refinement

**Table:**

Refinement on *F*^2^	Primary atom site location: structure-invariant direct methods
Least-squares matrix: full	Secondary atom site location: difference Fourier map
*R*[*F*^2^ > 2σ(*F*^2^)] = 0.035	Hydrogen site location: inferred from neighbouring sites
*wR*(*F*^2^) = 0.102	H-atom parameters constrained
*S* = 1.07	*w* = 1/[σ^2^(*F*_o_^2^) + (0.0559*P*)^2^ + 3.028*P*] where *P* = (*F*_o_^2^ + 2*F*_c_^2^)/3
6217 reflections	(Δ/σ)_max_ = 0.001
302 parameters	Δρ_max_ = 0.50 e Å^−^^3^
0 restraints	Δρ_min_ = −0.49 e Å^−^^3^

### Special details

**Table:**

Geometry. All e.s.d.'s (except the e.s.d. in the dihedral angle between two l.s. planes) are estimated using the full covariance matrix. The cell e.s.d.'s are taken into account individually in the estimation of e.s.d.'s in distances, angles and torsion angles; correlations between e.s.d.'s in cell parameters are only used when they are defined by crystal symmetry. An approximate (isotropic) treatment of cell e.s.d.'s is used for estimating e.s.d.'s involving l.s. planes.
Refinement. Refinement of *F*^2^ against ALL reflections. The weighted *R*-factor *wR* and goodness of fit *S* are based on *F*^2^, conventional *R*-factors *R* are based on *F*, with *F* set to zero for negative *F*^2^. The threshold expression of *F*^2^ > σ(*F*^2^) is used only for calculating *R*-factors(gt) *etc*. and is not relevant to the choice of reflections for refinement. *R*-factors based on *F*^2^ are statistically about twice as large as those based on *F*, and *R*- factors based on ALL data will be even larger.

### Fractional atomic coordinates and isotropic or equivalent isotropic displacement parameters (Å^2^)

**Table:**

	*x*	*y*	*z*	*U*_iso_*/*U*_eq_	
S1	0.196829 (15)	0.535633 (16)	0.521723 (15)	0.01859 (7)	
O1	0.19376 (6)	0.62050 (5)	0.55125 (6)	0.02835 (18)	
O2	0.18217 (5)	0.52321 (5)	0.43604 (5)	0.02233 (16)	
O3	0.22779 (6)	0.34464 (6)	0.47823 (6)	0.02816 (18)	
O4	0.09973 (6)	0.31848 (6)	0.39337 (6)	0.03011 (19)	
O5	0.29214 (5)	0.50520 (6)	0.69463 (5)	0.02743 (18)	
O6	0.65207 (5)	0.33795 (5)	0.66348 (5)	0.02148 (16)	
N1	0.29218 (5)	0.49756 (6)	0.56180 (5)	0.01930 (17)	
H1N	0.3228	0.4857	0.5294	0.023*	
N2	0.40259 (5)	0.44268 (6)	0.66704 (5)	0.01867 (17)	
H2N	0.4227	0.4320	0.7194	0.022*	
N3	0.52875 (5)	0.38975 (6)	0.66484 (5)	0.01831 (17)	
N4	0.55747 (6)	0.36370 (6)	0.53872 (5)	0.01880 (17)	
N5	0.42372 (5)	0.41857 (6)	0.54018 (5)	0.01753 (16)	
N6	0.45192 (6)	0.39316 (6)	0.41973 (5)	0.02194 (18)	
C1	0.12686 (6)	0.46904 (7)	0.55453 (7)	0.02002 (19)	
C2	0.10617 (7)	0.38937 (7)	0.51509 (7)	0.0220 (2)	
C3	0.04287 (8)	0.34016 (8)	0.52991 (8)	0.0295 (2)	
H3	0.0281	0.2869	0.5030	0.035*	
C4	0.00129 (8)	0.36899 (9)	0.58398 (9)	0.0323 (3)	
H4	−0.0426	0.3358	0.5934	0.039*	
C5	0.02355 (7)	0.44542 (9)	0.62381 (8)	0.0305 (3)	
H5	−0.0049	0.4634	0.6614	0.037*	
C6	0.08655 (7)	0.49787 (8)	0.61108 (7)	0.0252 (2)	
C7	0.15278 (7)	0.35064 (7)	0.46116 (7)	0.0229 (2)	
C8	0.13986 (9)	0.27562 (9)	0.34038 (9)	0.0349 (3)	
H8A	0.0972	0.2494	0.2949	0.052*	
H8B	0.1774	0.2312	0.3710	0.052*	
H8C	0.1722	0.3172	0.3193	0.052*	
C9	0.10498 (8)	0.57997 (10)	0.65897 (9)	0.0352 (3)	
H9A	0.1656	0.5858	0.6835	0.053*	
H9B	0.0773	0.5791	0.7020	0.053*	
H9C	0.0841	0.6285	0.6226	0.053*	
C10	0.32549 (6)	0.48361 (7)	0.64409 (6)	0.01966 (19)	
C11	0.45251 (6)	0.41618 (6)	0.62069 (6)	0.01614 (18)	
C12	0.57646 (6)	0.36447 (6)	0.61875 (6)	0.01739 (18)	
C13	0.47864 (7)	0.39181 (6)	0.50082 (6)	0.01759 (18)	
C14	0.36494 (8)	0.41607 (8)	0.37767 (7)	0.0259 (2)	
H14A	0.3530	0.4731	0.3951	0.039*	
H14B	0.3273	0.3743	0.3911	0.039*	
H14C	0.3564	0.4161	0.3188	0.039*	
C15	0.50356 (9)	0.36299 (8)	0.37022 (7)	0.0279 (2)	
H15A	0.5611	0.3547	0.4048	0.042*	
H15B	0.5030	0.4054	0.3281	0.042*	
H15C	0.4813	0.3086	0.3445	0.042*	
C16	0.71016 (7)	0.31386 (7)	0.62056 (7)	0.0224 (2)	
H16A	0.7187	0.3614	0.5858	0.027*	
H16B	0.6896	0.2633	0.5859	0.027*	
C17	0.78997 (8)	0.29349 (10)	0.68535 (9)	0.0334 (3)	
F1	0.84761 (5)	0.26665 (7)	0.65023 (7)	0.0484 (2)	
F2	0.78026 (6)	0.23027 (8)	0.73382 (7)	0.0546 (3)	
F3	0.82179 (6)	0.36048 (8)	0.73076 (7)	0.0570 (3)	

### Atomic displacement parameters (Å^2^)

**Table:**

	*U*^11^	*U*^22^	*U*^33^	*U*^12^	*U*^13^	*U*^23^
S1	0.01670 (12)	0.01668 (12)	0.02069 (13)	0.00019 (8)	0.00281 (9)	0.00071 (8)
O1	0.0256 (4)	0.0188 (4)	0.0389 (5)	0.0006 (3)	0.0068 (3)	−0.0046 (3)
O2	0.0207 (4)	0.0242 (4)	0.0199 (4)	0.0001 (3)	0.0026 (3)	0.0051 (3)
O3	0.0252 (4)	0.0237 (4)	0.0338 (5)	0.0012 (3)	0.0060 (3)	−0.0034 (3)
O4	0.0294 (4)	0.0285 (4)	0.0293 (4)	−0.0035 (3)	0.0038 (3)	−0.0060 (3)
O5	0.0214 (4)	0.0398 (5)	0.0203 (4)	0.0045 (3)	0.0048 (3)	−0.0065 (3)
O6	0.0160 (3)	0.0298 (4)	0.0192 (3)	0.0026 (3)	0.0060 (3)	0.0009 (3)
N1	0.0155 (4)	0.0234 (4)	0.0175 (4)	0.0012 (3)	0.0025 (3)	−0.0011 (3)
N2	0.0165 (4)	0.0263 (4)	0.0124 (3)	0.0017 (3)	0.0028 (3)	−0.0007 (3)
N3	0.0153 (4)	0.0251 (4)	0.0139 (4)	0.0002 (3)	0.0033 (3)	0.0004 (3)
N4	0.0213 (4)	0.0197 (4)	0.0164 (4)	−0.0022 (3)	0.0070 (3)	−0.0011 (3)
N5	0.0191 (4)	0.0192 (4)	0.0130 (4)	−0.0012 (3)	0.0028 (3)	−0.0003 (3)
N6	0.0271 (4)	0.0253 (4)	0.0133 (4)	−0.0033 (3)	0.0058 (3)	−0.0003 (3)
C1	0.0165 (4)	0.0220 (5)	0.0199 (4)	0.0002 (3)	0.0027 (3)	0.0032 (4)
C2	0.0202 (5)	0.0199 (5)	0.0240 (5)	0.0000 (4)	0.0036 (4)	0.0040 (4)
C3	0.0245 (5)	0.0237 (5)	0.0385 (6)	−0.0032 (4)	0.0062 (5)	0.0078 (5)
C4	0.0222 (5)	0.0360 (6)	0.0389 (7)	0.0002 (5)	0.0092 (5)	0.0161 (5)
C5	0.0203 (5)	0.0442 (7)	0.0276 (6)	0.0058 (5)	0.0080 (4)	0.0104 (5)
C6	0.0185 (5)	0.0337 (6)	0.0222 (5)	0.0036 (4)	0.0038 (4)	0.0017 (4)
C7	0.0252 (5)	0.0152 (4)	0.0266 (5)	−0.0019 (4)	0.0048 (4)	0.0014 (4)
C8	0.0402 (7)	0.0314 (6)	0.0322 (6)	−0.0034 (5)	0.0091 (5)	−0.0095 (5)
C9	0.0251 (6)	0.0480 (8)	0.0327 (6)	0.0036 (5)	0.0089 (5)	−0.0129 (6)
C10	0.0163 (4)	0.0229 (5)	0.0180 (4)	−0.0014 (4)	0.0023 (3)	−0.0032 (4)
C11	0.0156 (4)	0.0177 (4)	0.0143 (4)	−0.0022 (3)	0.0031 (3)	−0.0003 (3)
C12	0.0169 (4)	0.0181 (4)	0.0172 (4)	−0.0022 (3)	0.0052 (3)	0.0001 (3)
C13	0.0222 (5)	0.0159 (4)	0.0147 (4)	−0.0042 (3)	0.0054 (3)	−0.0005 (3)
C14	0.0293 (5)	0.0301 (5)	0.0143 (4)	−0.0027 (4)	0.0000 (4)	0.0014 (4)
C15	0.0381 (6)	0.0310 (6)	0.0176 (5)	−0.0032 (5)	0.0131 (4)	−0.0024 (4)
C16	0.0195 (4)	0.0237 (5)	0.0270 (5)	0.0007 (4)	0.0118 (4)	0.0007 (4)
C17	0.0203 (5)	0.0438 (7)	0.0387 (7)	0.0052 (5)	0.0127 (5)	0.0088 (5)
F1	0.0268 (4)	0.0632 (6)	0.0641 (6)	0.0147 (4)	0.0270 (4)	0.0148 (5)
F2	0.0356 (5)	0.0737 (7)	0.0595 (6)	0.0213 (5)	0.0215 (4)	0.0405 (5)
F3	0.0273 (4)	0.0810 (8)	0.0545 (6)	−0.0069 (4)	−0.0011 (4)	−0.0191 (5)

### Geometric parameters (Å, °)

**Table:**

S1—O1	1.4274 (9)	C2—C7	1.5020 (16)
S1—O2	1.4337 (8)	C3—C4	1.389 (2)
S1—N1	1.6507 (9)	C3—H3	0.9500
S1—C1	1.7765 (11)	C4—C5	1.374 (2)
O3—C7	1.2044 (15)	C4—H4	0.9500
O4—C7	1.3385 (14)	C5—C6	1.4016 (17)
O4—C8	1.4444 (16)	C5—H5	0.9500
O5—C10	1.2094 (13)	C6—C9	1.5076 (19)
O6—C12	1.3381 (12)	C8—H8A	0.9800
O6—C16	1.4311 (12)	C8—H8B	0.9800
N1—C10	1.3771 (13)	C8—H8C	0.9800
N1—H1N	0.8800	C9—H9A	0.9800
N2—C11	1.3753 (13)	C9—H9B	0.9800
N2—C10	1.3884 (13)	C9—H9C	0.9800
N2—H2N	0.8800	C14—H14A	0.9800
N3—C12	1.3378 (13)	C14—H14B	0.9800
N3—C11	1.3429 (12)	C14—H14C	0.9800
N4—C12	1.3179 (13)	C15—H15A	0.9800
N4—C13	1.3602 (14)	C15—H15B	0.9800
N5—C11	1.3258 (12)	C15—H15C	0.9800
N5—C13	1.3561 (13)	C16—C17	1.5000 (17)
N6—C13	1.3333 (13)	C16—H16A	0.9900
N6—C15	1.4566 (15)	C16—H16B	0.9900
N6—C14	1.4656 (15)	C17—F3	1.3216 (19)
C1—C6	1.4090 (16)	C17—F2	1.3332 (16)
C1—C2	1.4122 (15)	C17—F1	1.3449 (15)
C2—C3	1.3906 (16)		
			
O1—S1—O2	118.56 (5)	H8A—C8—H8C	109.5
O1—S1—N1	108.54 (5)	H8B—C8—H8C	109.5
O2—S1—N1	103.49 (5)	C6—C9—H9A	109.5
O1—S1—C1	109.68 (5)	C6—C9—H9B	109.5
O2—S1—C1	108.44 (5)	H9A—C9—H9B	109.5
N1—S1—C1	107.50 (5)	C6—C9—H9C	109.5
C7—O4—C8	114.32 (10)	H9A—C9—H9C	109.5
C12—O6—C16	117.02 (8)	H9B—C9—H9C	109.5
C10—N1—S1	122.42 (8)	O5—C10—N1	124.11 (10)
C10—N1—H1N	118.8	O5—C10—N2	120.60 (10)
S1—N1—H1N	118.8	N1—C10—N2	115.29 (9)
C11—N2—C10	130.10 (9)	N5—C11—N3	126.34 (9)
C11—N2—H2N	114.9	N5—C11—N2	120.00 (9)
C10—N2—H2N	114.9	N3—C11—N2	113.67 (8)
C12—N3—C11	112.81 (8)	N4—C12—N3	128.15 (9)
C12—N4—C13	113.58 (9)	N4—C12—O6	119.67 (9)
C11—N5—C13	114.85 (9)	N3—C12—O6	112.18 (9)
C13—N6—C15	122.09 (10)	N6—C13—N5	116.63 (9)
C13—N6—C14	119.97 (9)	N6—C13—N4	119.11 (9)
C15—N6—C14	117.57 (9)	N5—C13—N4	124.26 (9)
C6—C1—C2	120.93 (10)	N6—C14—H14A	109.5
C6—C1—S1	121.56 (9)	N6—C14—H14B	109.5
C2—C1—S1	117.16 (8)	H14A—C14—H14B	109.5
C3—C2—C1	119.53 (11)	N6—C14—H14C	109.5
C3—C2—C7	116.72 (10)	H14A—C14—H14C	109.5
C1—C2—C7	123.62 (10)	H14B—C14—H14C	109.5
C4—C3—C2	119.96 (12)	N6—C15—H15A	109.5
C4—C3—H3	120.0	N6—C15—H15B	109.5
C2—C3—H3	120.0	H15A—C15—H15B	109.5
C5—C4—C3	120.04 (11)	N6—C15—H15C	109.5
C5—C4—H4	120.0	H15A—C15—H15C	109.5
C3—C4—H4	120.0	H15B—C15—H15C	109.5
C4—C5—C6	122.53 (12)	O6—C16—C17	105.26 (9)
C4—C5—H5	118.7	O6—C16—H16A	110.7
C6—C5—H5	118.7	C17—C16—H16A	110.7
C5—C6—C1	116.95 (11)	O6—C16—H16B	110.7
C5—C6—C9	117.13 (11)	C17—C16—H16B	110.7
C1—C6—C9	125.92 (11)	H16A—C16—H16B	108.8
O3—C7—O4	124.01 (11)	F3—C17—F2	108.69 (13)
O3—C7—C2	124.89 (11)	F3—C17—F1	107.18 (11)
O4—C7—C2	110.97 (10)	F2—C17—F1	106.45 (11)
O4—C8—H8A	109.5	F3—C17—C16	112.73 (12)
O4—C8—H8B	109.5	F2—C17—C16	112.14 (11)
H8A—C8—H8B	109.5	F1—C17—C16	109.34 (11)
O4—C8—H8C	109.5		
			
O1—S1—N1—C10	64.66 (10)	S1—N1—C10—O5	−7.42 (16)
O2—S1—N1—C10	−168.53 (9)	S1—N1—C10—N2	173.18 (8)
C1—S1—N1—C10	−53.90 (10)	C11—N2—C10—O5	−177.04 (11)
O1—S1—C1—C6	−8.55 (11)	C11—N2—C10—N1	2.38 (16)
O2—S1—C1—C6	−139.44 (9)	C13—N5—C11—N3	−0.51 (15)
N1—S1—C1—C6	109.28 (9)	C13—N5—C11—N2	179.32 (9)
O1—S1—C1—C2	164.73 (8)	C12—N3—C11—N5	0.02 (15)
O2—S1—C1—C2	33.84 (10)	C12—N3—C11—N2	−179.81 (9)
N1—S1—C1—C2	−77.44 (9)	C10—N2—C11—N5	−8.96 (17)
C6—C1—C2—C3	2.50 (16)	C10—N2—C11—N3	170.89 (10)
S1—C1—C2—C3	−170.82 (9)	C13—N4—C12—N3	−0.72 (15)
C6—C1—C2—C7	−173.24 (10)	C13—N4—C12—O6	179.68 (9)
S1—C1—C2—C7	13.44 (14)	C11—N3—C12—N4	0.66 (15)
C1—C2—C3—C4	−0.85 (17)	C11—N3—C12—O6	−179.72 (9)
C7—C2—C3—C4	175.18 (11)	C16—O6—C12—N4	3.04 (14)
C2—C3—C4—C5	−1.03 (19)	C16—O6—C12—N3	−176.62 (9)
C3—C4—C5—C6	1.33 (19)	C15—N6—C13—N5	−177.58 (10)
C4—C5—C6—C1	0.28 (17)	C14—N6—C13—N5	−4.83 (14)
C4—C5—C6—C9	−179.79 (12)	C15—N6—C13—N4	1.92 (15)
C2—C1—C6—C5	−2.19 (16)	C14—N6—C13—N4	174.67 (9)
S1—C1—C6—C5	170.84 (9)	C11—N5—C13—N6	179.90 (9)
C2—C1—C6—C9	177.89 (11)	C11—N5—C13—N4	0.43 (14)
S1—C1—C6—C9	−9.08 (16)	C12—N4—C13—N6	−179.33 (9)
C8—O4—C7—O3	−0.53 (17)	C12—N4—C13—N5	0.12 (14)
C8—O4—C7—C2	−176.61 (10)	C12—O6—C16—C17	176.05 (10)
C3—C2—C7—O3	−127.73 (13)	O6—C16—C17—F3	−63.38 (13)
C1—C2—C7—O3	48.12 (17)	O6—C16—C17—F2	59.68 (14)
C3—C2—C7—O4	48.31 (14)	O6—C16—C17—F1	177.50 (10)
C1—C2—C7—O4	−135.84 (11)		

### Hydrogen-bond geometry (Å, °)

**Table:**

*D*—H···*A*	*D*—H	H···*A*	*D*···*A*	*D*—H···*A*
N1—H1N···N5	0.88	1.95	2.6410 (13)	135
N2—H2N···N3^i^	0.88	2.03	2.8998 (12)	172
C9—H9A···O5	0.98	2.42	3.2253 (16)	139
C14—H14B···O3	0.98	2.59	3.4363 (16)	145
C14—H14C···O5^ii^	0.98	2.42	3.2637 (14)	143
C15—H15A···N4	0.98	2.33	2.7695 (15)	107
C16—H16A···O2^iii^	0.99	2.55	3.4209 (14)	147
C16—H16B···O1^iv^	0.99	2.32	3.2325 (14)	153

Symmetry codes: (i) −*x*+1, *y*, −*z*+3/2; (ii) *x*, −*y*+1, *z*−1/2; (iii) −*x*+1, −*y*+1, −*z*+1; (iv) *x*+1/2, *y*−1/2, *z*.
